# An Improved Deep Residual Network-Based Semantic Simultaneous Localization and Mapping Method for Monocular Vision Robot

**DOI:** 10.1155/2020/7490840

**Published:** 2020-02-10

**Authors:** Jianjun Ni, Tao Gong, Yafei Gu, Jinxiu Zhu, Xinnan Fan

**Affiliations:** ^1^College of IOT Engineering, Hohai University, Changzhou 213022, China; ^2^Jiangsu Universities and Colleges Key Laboratory of Special Robot Technology, Hohai University, Changzhou 213022, China

## Abstract

The robot simultaneous localization and mapping (SLAM) is a very important and useful technology in the robotic field. However, the environmental map constructed by the traditional visual SLAM method contains little semantic information, which cannot satisfy the needs of complex applications. The semantic map can deal with this problem efficiently, which has become a research hot spot. This paper proposed an improved deep residual network- (ResNet-) based semantic SLAM method for monocular vision robots. In the proposed approach, an improved image matching algorithm based on feature points is presented, to enhance the anti-interference ability of the algorithm. Then, the robust feature point extraction method is adopted in the front-end module of the SLAM system, which can effectively reduce the probability of camera tracking loss. In addition, the improved key frame insertion method is introduced in the visual SLAM system to enhance the stability of the system during the turning and moving of the robot. Furthermore, an improved ResNet model is proposed to extract the semantic information of the environment to complete the construction of the semantic map of the environment. Finally, various experiments are conducted and the results show that the proposed method is effective.

## 1. Introduction

With the rapid development of computer technology and sensor technology, the research and application of robots have reached a new height [1–4]. For mobile robots, when facing an unknown environment, they need to use their own sensor devices to sense the surrounding environment, build an environment map by moving, and determine their positions in the map; this is called robot simultaneous localization and mapping (SLAM) problem [5–7]. Its basic principle is that the mobile robot can sense its location environment, establish a continuous environment map, and complete its accurate positioning on the map. In recent years, SLAM technology of robot has made some achievements, such as SLAM based on laser radar and sonar, SLAM based on robot vision, and so on [8, 9]. The visual SLAM is one of the most used SLAM technologies, which can be divided into monocular SLAM, binocular SLAM, and multivision SLAM [10, 11].

Although the traditional SLAM maps can help robots to locate themselves, they lack the understanding of the environment required for specific tasks, namely, semantic information. The robot semantic SLAM technology can deal with this problem, so more and more research has focused on the robot semantic SLAM method. For example, Civera et al. [12] combined the target recognition method with the SLAM method of monocular vision based on extended Kalman filtering and ran the two threads simultaneously to achieve semantic SLAM. Bowman et al. [13] formulated the problem of related optimization of sensor states and semantic landmarks and proposed the semantic SLAM algorithm of probability data association. Günther et al. [14] presented an approach to create a semantic map of an indoor environment incrementally and in closed loop, based on a semantic model about furniture objects and a series of 3D point clouds captured by a mobile robot using an RGB-D camera. These methods above can deal with the semantic SLAM problem efficiently; however, there are still many problems to be solved. For example, the object recognition needs lots of training data and selects an appropriate classifier to identify objects from various angles. Also, the requirements on the real-time and readability performance of the semantic SLAM are difficult to meet at the same time.

Recently, more and more deep learning-based methods have been proposed to deal with the robot semantic SLAM problem; the basic idea of these methods is that the traditional SLAM method is combined with deep learning technology. For example, Sunderhauf et al. [15] proposed a semantic map construction method oriented to rich objects, which applied convolutional neural network (CNN) to construct a three-dimensional semantic map based on the three-dimensional point cloud of the objects. Li et al. [16] proposed a semantic pixelwise mapping system, which includes a novel spatiotemporal deep neural network for semantic segmentation and a SLAM algorithm for 3D point cloud map. McCormac et al. [17] constructed dense three-dimensional semantic maps using convolutional neural network, which used the ElasticFusion as a SLAM system and CNN for semantic segmentation. The deep learning-based semantic map construction method for the robotic visual SLAM is a research hot spot and developing trend. But, there are still many problems to be solved, such as the computational efficiency and mapping precision. In this paper, an improved semantic map construction method for monocular vision robot is presented, which is a novel integrated method.

The main contributions of this paper are summarized as follows: (1) an improved image matching method based on feature points is proposed to deal with the problem of low image matching accuracy and poor anti-interference performance, by using an adaptive FAST corner detection method and a double constraint strategy; (2) the visual SLAM method is studied and improved to deal with the problem of the poor tracking ability and low positioning accuracy in the traditional visual SLAM method, by using the proposed image matching method and key frame selection mechanism; (3) an improved deep learning-based semantic map construction method is presented, which is based on the ResNet residual neural network model. Finally, in order to verify the feasibility of the proposed method, several experiments are designed to construct the semantic map of the environment and the results are analyzed. Experimental results show that the proposed method is effective and feasible.

This paper is organized as follows.  Section 2 presents the proposed approach. The experiments under various environments are given in Section 3.  Section 4 discusses the performance of the proposed approach. Finally, the conclusions are given in Section 5.

## 2. The Proposed Approach

**Figure 1:**
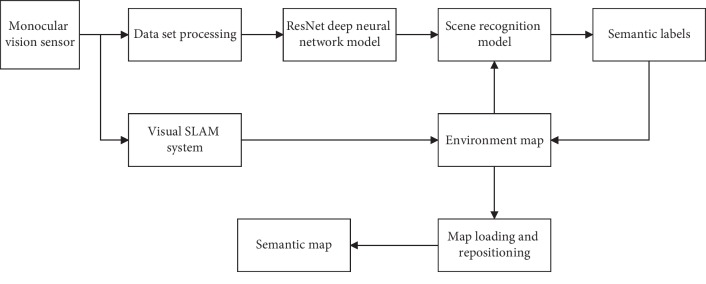
The frame of the proposed semantic SLAM method.

In this paper, the problem of semantic map construction method is studied, and a novel integrated approach is proposed to deal with this problem. The basic idea of the proposed method is to combine the visual SLAM method with deep learning to construct environmental semantic map, which mainly includes image matching algorithm, monocular visual SLAM method, and semantic map construction method. In the proposed method, the main work of the data set processing before the scene recognition by the ResNet network is the scene image tagging. The frame of the proposed method is shown in Figure 1 and will be introduced in detail as follows.

### 2.1. Image Matching Algorithm

In the visual SLAM, the image matching is a key process. In the feature extraction and matching process, the commonly used methods include scale-invariant feature transform algorithm (SIFT), speed-up robust features algorithm (SURF), oriented FAST and rotated BRIEF (ORB), and so on [18–20]. The feature points extracted by SIFT are very stable and remain unchanged in the case of scale change, rotation, and contrast difference of the image. However, the SIFT algorithm has the disadvantage of slow running speed. The SURF algorithm is based on the SIFT algorithm, which can effectively identify the image feature points, and inherits the advantages of the SIFT method. The ORB algorithm is essentially a combination of the improved FAST algorithm and the BRIEF feature point description algorithm. Experimental research shows that ORB is an order of magnitude faster than SURF in terms of execution efficiency and two orders of magnitude faster than SIFT. Therefore, an improved image matching method based on ORB algorithm (defined as I-ORB) is proposed in this paper, to improve the image matching performance, where an adaptive threshold selection method based on image contrast is used to replace the fixed experience value in the traditional algorithm for feature point extraction, and a double constraint strategy based on geometric features and affine invariant constraints is used to eliminate mismatched feature points and to improve the accuracy of image matching. The improvements are introduced in details as follows.

In the process of feature point extraction based on the FAST method, it is realized by comparing the grayscale value of candidate pixel point and its surrounding pixel points. The calculating strategy is shown as follows:

(1) $$\begin{array}{c} f_{\det}=\left\{\begin{array}{cc} 1, & \left|I_{x}-I_{C}\right|\geq t,\\ 0, & \left|I_{x}-I_{C}\right|<t, \end{array}\right.\\ \underset{x\in \text{circle}\left(C\right)}{\sum }f_{\det}\left(I_{x},I_{C}\right)\geq N, \end{array}$$

where *I*_*C*_ is the grayscale value of candidate pixel point *C*, *I*_*x*_ is the grayscale value of the surrounding pixels (denoted by circle(*C*)), *t* is the threshold, and *N* is a predefined value. If there are more than *N* consecutive pixels and the absolute difference values between their gray value and the gray value of the central pixel *I*_*C*_ is greater than or equal to the threshold *t*, the candidate pixel *I*_*C*_ can be considered as the feature point.

In the traditional FAST algorithm described above, the threshold *t* is fixed, which has poor anti-interference performance. To deal with this problem, an adaptive FAST corner detection threshold is proposed, where the threshold value *t* is calculated as follows:

(2) $$\begin{array}{c} t=\alpha \cdot \left(\frac{1}{n}\overset{n}{\underset{i=1}{\sum }}{\left(I\left(x_{i}\right)-I\left(\bar{x}\right)\right)}^{2}\right), \end{array}$$

where *α* is the scale coefficient, *I*(*x*_*i*_) is the gray value of each pixel of the image, $I\left(\bar{x}\right)$ is the gray mean of the image, and *n* is the total number of pixels of the image.

**Remark 1 .**

Although there is a parameter *α* in the equation of calculating *t*, it is different from the fixed value of the traditional FAST algorithm. Because the threshold *t* is calculated adaptively based on the actual image contrast and the parameter *α* is just an adjustment coefficient, which determines the number of feature points extracted from the image, it is easier to decide the value of *α* than to decide the value of *t* directly [21]. In this paper, the value of *α* is set as 0.01 by comprehensive consideration of the size of image and the computation complexity, based on the simple trial method. Also, the value of *α* can be obtained based on some intelligent optimization methods, which have been studied in other literature [22, 23].

In the image matching process, the traditional ORB algorithm uses the violent matching method to match the two images, which is very fast but of poor quality. In order to improve the accuracy of image matching, double constraints are proposed to the rough matching results of the ORB algorithm, namely, the geometric characteristic constraint and the affine invariant constraint. The details of the proposed method are as follows:

1. Making use of the geometric characteristic constraint for preliminary screening: after obtaining the rough matching results of the two images, the geometric characteristics of the images can be used as a constraint for preliminary screening [24]. Firstly, the sets of feature points obtained from rough matching results of two images are defined as *P* and *Q*, respectively. 〈*P*_1_, *Q*_1_〉 and 〈*P*_2_, *Q*_2_〉 are two groups of correct matching points obtained from matching two related images, according to the geometric characteristics of images. Then, some evaluation functions are defined:

(3) $$\begin{array}{c} W\left(i\right)=\overset{T}{\underset{j=1}{\sum }}\frac{r\left(i,j\right)}{1+D\left(i,j\right)},\\ D\left(i,j\right)=\frac{\left[d\left(P_{i},P_{j}\right)+d\left(Q_{i},Q_{j}\right)\right]}{2},\\ r\left(i,j\right)=\exp\left(-\frac{\left|d\left(P_{i},P_{j}\right)-d\left(Q_{i},Q_{j}\right)\right|}{D\left(i,j\right)}\right), \end{array}$$

  where *d*(*P*_*i*_, *P*_*j*_) represents the Euclidean distance of two feature points *P*_*i*_ and *P*_*j*_ in the same set; *r*(*i*, *j*) represents the similarity difference between feature points; and *T* is the number of the feature points obtained from the rough matching. The steps of the preliminary screening process based on the geometric characteristic constraint are as follows: (1) calculate all values of *W*(*i*); (2) calculate the standard deviations Std(*W*) of all *W*(*i*); (3) if *W*(*i*) > Std(*W*), it can be considered to be a correct matching point; otherwise, it is discarded as a false matching point.
(2) Making use of the affine invariant constraint for further screening: after the preliminary screening above, some feature points of mismatching are eliminated and the rest sets of feature matching points are redefined as *P*′ and *Q*′, and the matching results are further optimized through affine invariance constraint [25]. For a pair of matching feature points 〈*P*_*i*_′, *Q*_*i*_′〉, the Euclidean distance is used to find *k* feature points closest to point *P*_*i*_′ and point *Q*_*i*_′ in two sets, and they are placed in the subsets *p* and *q*. According to the affine invariance theorem for two related images, if 〈*P*_*i*_′, *Q*_*i*_′〉 is a pair of correct matching points, then the points in its subsets *p* and *q* should also match in pairs.

In order to reduce noise interference, the two points in the set that are closest and farthest from the current feature point are removed, and the *k* − 2 points in the middle are kept, namely,

(4) $$\begin{array}{c} \left\{\begin{array}{c} p=\left\{p_{1},p_{2},\ldots ,p_{k-2}\right\},\\ q=\left\{q_{1},q_{2},\ldots ,p_{k-2}\right\}. \end{array}\right. \end{array}$$

Here, the number of matching points existing in the subsets *p* and *q* is taken as affine invariant constraint item. And, the determination mechanism for the correct matching of feature points is as follows:

(5) $$\begin{array}{c} M\langle P_{i}^{\prime },Q_{i}^{\prime }\rangle =\left\{\begin{array}{cc} 1, & s>\left(\frac{k-2}{2}\right),\\ 0, & \text{otherwise,} \end{array}\right. \end{array}$$

where *M* is a flag to denote whether the feature points are a pair of correct matching points and *s* is the number of matching corresponding feature points in *p* and *q*.

### 2.2. Monocular Vision SLAM Algorithm

**Figure 2:**
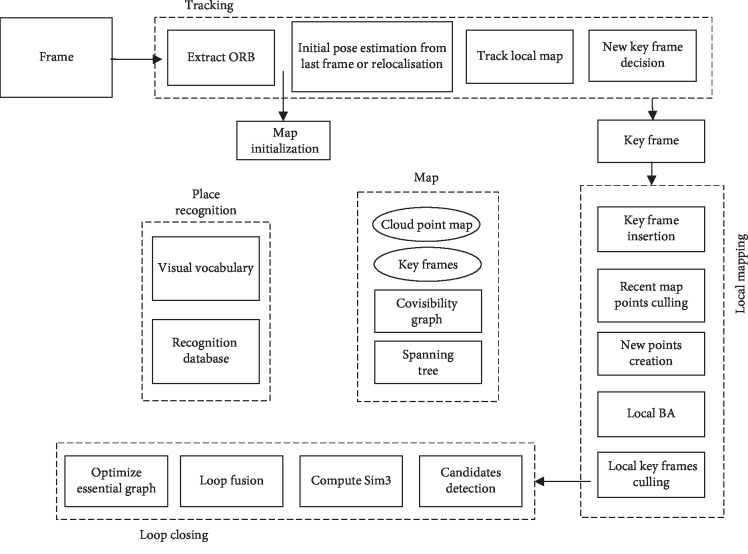
The frame of the ORB SLAM system for monocular vision robot.

In this paper, the SLAM system based on image feature points is constructed, which has good real-time performance and can operate in large, small, indoor, and outdoor environments. In this study, the unified ORB features are used to process image frames, which can avoid the time and space cost caused by feature point re-extraction effectively. In addition, because ORB features have certain illumination and rotation invariance, the robustness of the system is better [26, 27]. The system framework is shown in Figure 2, which is the same as the traditional ORB SLAM system [28]. In the proposed visual SLAM algorithm, its performance has been improved, by using the improved ORB feature extraction and matching scheme to improve the accuracy of pose estimation and an improved key frame selection method to enhance the stability of robot tracking during turning.

There are three main interrelated threads in the system, namely, camera pose tracking, local mapping, and loop closing detection. The location recognition module is mainly used for global repositioning and closed-loop detection of scenes. In camera pose tracking, the improved FAST feature point extraction algorithm (Section 2.1) is used to deal with the feature extraction and image feature matching problem. In initializing pose estimation, when the previous frame is successfully tracked, the same motion model can be used to calculate the current position of the camera and find out cloud points of the map observed in the previous frame. If tracking loss occurs, index technology is used to match the current image frame with previous key frames to find the most similar scene image. In addition, the random sampling consistency algorithm (RANSAC) is used to eliminate false matching points [29], and the PnP algorithm is used to calculate the relative position of the camera at the current moment [30]. Finally, bundle adjustment (BA) optimization is adopted to complete the initial pose estimation of the camera [31].

In the monocular vision SLAM algorithm, the key frame selection mechanism is an important part. In the general vision SLAM algorithm, the selection mechanism is mostly based on fixed rules, which cannot deal with the tracking lost problem caused by the rotation of robot. To deal with this problem, an improved key frame selection method based on robot rotation angle is proposed. Assuming that the robot rotates by an angle *θ* around the unit vector *U*=[*U*_*x*_, *U*_*y*_, *U*_*z*_]^*T*^ in world coordinates (where *U*_*x*_, *U*_*y*_,  and *U*_*z*_ represent the components of the unit vector on the *x*, *y* and *z* axes respectively), the basic mathematical equation of the four elements in the SLAM system can be expressed as

(6) $$\begin{array}{c} R={\left[\cos\frac{\theta }{2},U_{x}\sin\frac{\theta }{2},U_{y}\sin\frac{\theta }{2},U_{z}\sin\frac{\theta }{2}\right]}^{T}. \end{array}$$

The turning process of the robot can be regarded as rotating around the *z*-axis. In this paper, the absolute value Δ*θ* of the rotation angle difference between two adjacent frames of images *i* and *j* is calculated to represent the rotation angle of the robot, which can be expressed as

(7) $$\begin{array}{c} \Delta \theta_{\left(i,j\right)}=\overset{j-1}{\underset{k=i}{\sum }}\Delta \theta_{\left(k,k+1\right)}. \end{array}$$

**Figure 3:**
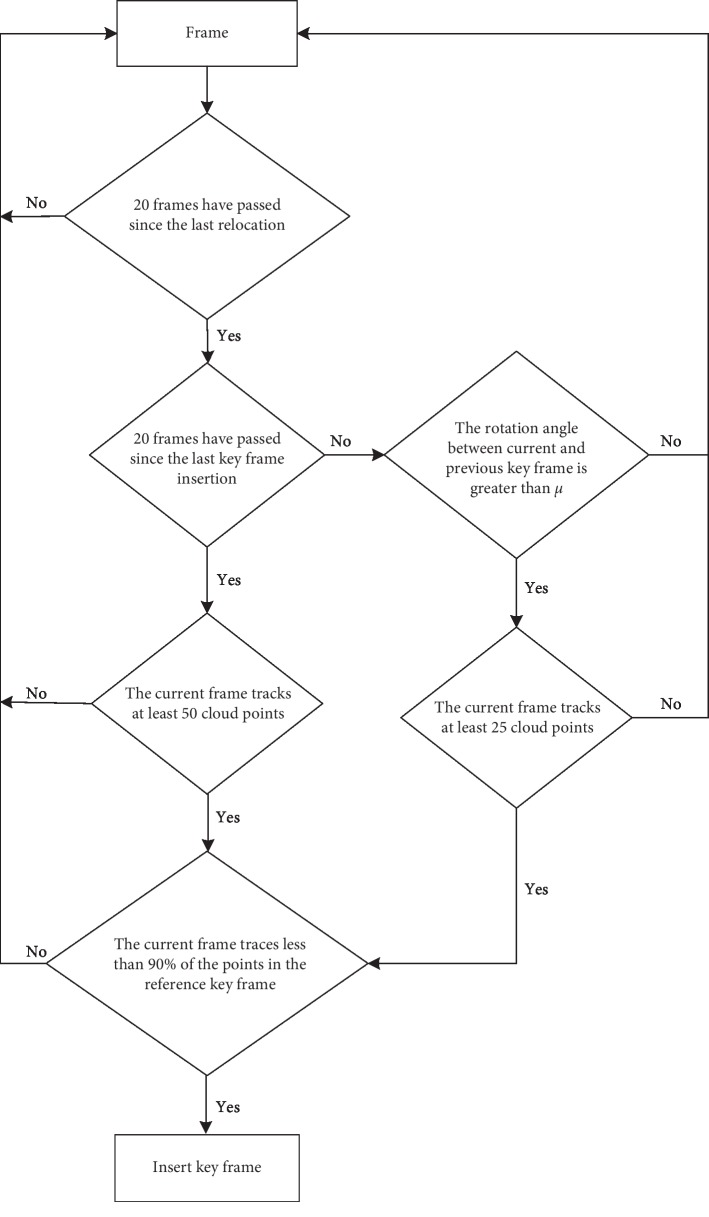
The flowchart of the improved key frame insertion mechanism.

In order to enhance the tracking stability of the robot during turning, an improved key frame selection method is presented in this paper, and its specific flow is shown in Figure 3. The detail work mechanism of the improved key frame selection is as follows. When the mobile robot rotates to a certain angle, the key frame needs to be inserted as soon as possible to ensure that the tracking is not lost. Namely, if there are less than 20 frames from the last key frame inserted and the rotation of the camera on the current frame is greater than a threshold *μ* between the last key frame, a new key frame should be inserted. In this paper, the range of *μ* is set as *μ* ∈ [7,12]. Considering the difficulty of matching and tracking between two frames of images in the process of camera rotation, it is only required that the current frame can track more than 25 cloud points when the rotation condition is satisfied. Finally, based on the improved key frame selection algorithm, local map construction and closed loop detection are completed. In this key frame insertion method, the concrete values for the parameters are obtained by experience, which have been used and proven to be effective in other literature [26, 28].

### 2.3. Semantic Map Construction Method

To realize the scene classification and recognition, ResNet deep residual network is adopted in this paper [32]. Although there are lots of methods such as SVM and fuzzy k-NN, which have been successfully applied in image classification, the performance of these traditional image classification methods cannot satisfy the requirements of the semantic SLAM system [33, 34]. So, the deep learning method is adopted in the proposed semantic SLAM system, which is suitable for the complex semantic recognition. The main reason of using ResNet deep residual network is that it can solve the problem about increasing training errors when deepening the depth of the neural network and improve the accuracy of the model effectively. With the increasing of network depth, the accuracy will not decline. So, the ResNet deep residual network breaks the previous layer number constraint of the convolutional neural network and provides feasibility for the extraction and classification of deeper semantic features [35, 36].

In this paper, a 50-layer residual neural network (ResNet50) model is built to classify the collected scene images and add semantic information to the environment map. Firstly, the scene images are collected and classified. And then, they are used to train the ResNet residual network model to classify different scenes. At last, the trained ResNet residual network is used in the semantic SLAM system, to complete the construction of the semantic map of the environment, by classifying all of the key frames obtained in the process of the visual SLAM.

**Figure 4:**
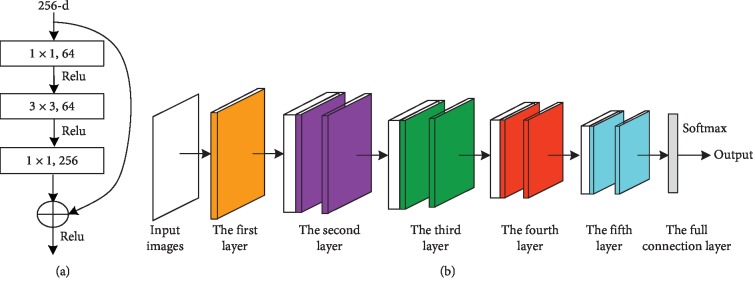
The structure diagram of ResNet network model: (a) the model structure of ResNet and (b) the overall structure of the 50-layer ResNet.

The model structure of the ResNet residual network is shown in Figure 4(a), which concatenates the convolution kernel of 1*∗*1, 3*∗*3, and 1*∗*1. This structure not only guarantees the accuracy of the algorithm but also greatly reduces the number of calculation and parameters. The overall structure of the 50-layer ResNet convolutional neural network selected in this paper is shown in Figure 4(b).

The details of the proposed ResNet residual neural network are introduced as follows [32, 37]. Input layer: the inputs of the ResNet are color images of the real scenes, and the size of the input image is 224*∗*224*∗*3. Layer 1 (the first convolution layer): the convolution kernel is 7*∗*7*∗*64. Layer 2 (the maximum pool layer and the second convolution layer): the pool core of the pool layer is 3*∗*3, and the convolution layer contains three module units, which are made up of three convolution cores, namely, 1*∗*1*∗*64, 3*∗*3*∗*64, and 1*∗*1*∗*256. Layer 3 (the third convolution layer): it contains four module units, and each module is composed of three convolution cores, namely, 1*∗*1*∗*128, 3*∗*3*∗*128, and 1*∗*1*∗*512. Layer 4 (the fourth convolution layer): it contains six module units, and each module is made up of three convolution cores, namely, 1*∗*1*∗*256, 3*∗*3*∗*256, and 1*∗*1*∗*1024. Layer 5 (the fifth convolution layer and the average pool layer): the convolution layer contains three module units, and each module is made up of three convolution cores, namely, 1*∗*1*∗*512, 3*∗*3*∗*512, and 1*∗*1*∗*2048. The pool core of the pool layer is 7*∗*7. Layer 6 (the full connection layer): it connects all the features and sends the outputs to the classifier at a lower level. Output layer: the loss function in this paper is Softmax function.

In order to construct the environment semantic map for human-computer interaction successfully, the semantic classification information *c* is added to the key frames in the map, where the value of *c* is an integer between 1 to *L* and *L* is the number of scenes. At the same time, the activity value of classification *A* is added to each key frame. Therefore, the semantic key frame can be expressed as follows:

(8) $$\begin{array}{c} {\text{Frame}}_{c}=\left\{T_{\text{iw}},V,F,c,A\right\}, \end{array}$$

where *T*_iw_ is the camera pose, *V* is the internal parameters of camera, and *F* denotes all ORB features.

**Figure 5:**
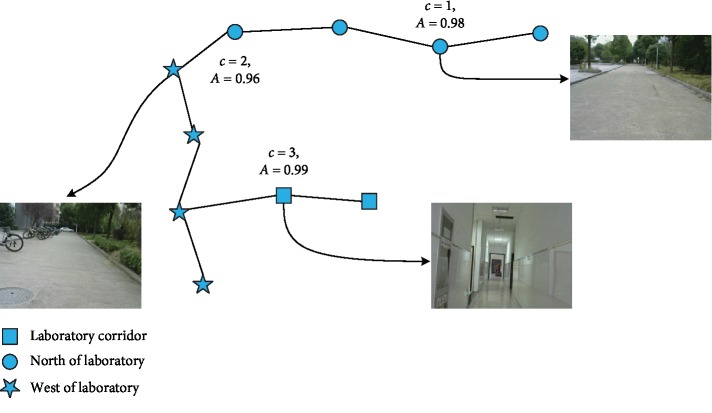
An example of semantic map construction.

Then, the trained ResNet residual network model is used to classify all of the key frames of the environment map, which are obtained by the proposed monocular vision SLAM algorithm (Section 2.2). The output of the network is the probability that each key frame belongs to each scene category. If the maximum probability value of the *c*-th scene is *A*, it means that the key frame image belongs to this scene. An example of this process is shown in Figure 5.

## 3. Experiments

In order to verify the feasibility of the proposed method, two experiments are designed to construct the semantic map of the environment. The amigo pioneer robot was used as the experimental platform in the experiment, and a monocular camera was equipped as the vision sensor to provide images. The main configurations of the laptop used in the experiment are as follows: 8G memory and 3.5 GHz CPU. The framework of Keras and TensorFlow was used for working with the deep convolutional ResNet in this paper.

**Figure 6:**
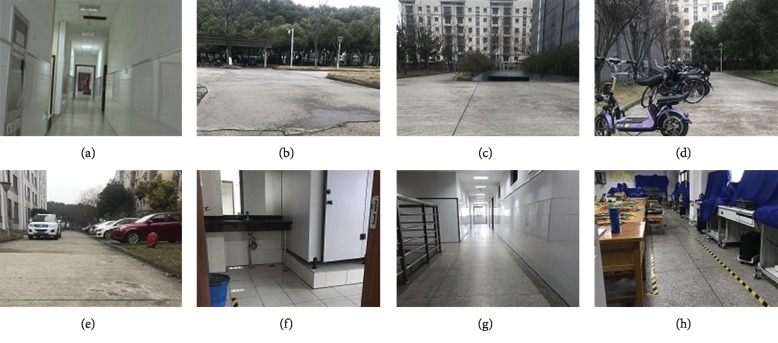
The main scenes in these experiments: (a) internal passage; (b) north; (c) west; (d) south; (e) east; (f) toilet; (g) corridor; (h) laboratory.

In this paper, the indoor and outdoor scenes of a comprehensive experimental building are selected as the experimental environment for semantic map creation. The main scenes are shown in Figure 6. In the experiments of this study, the images of different scenes are used to train the ResNet neural network. For each scene, 600 images are collected, 90% of them are used to train the ResNet, and 10% of them are used for test.

### 3.1. The Experiment of Indoor Environment

**Figure 7:**
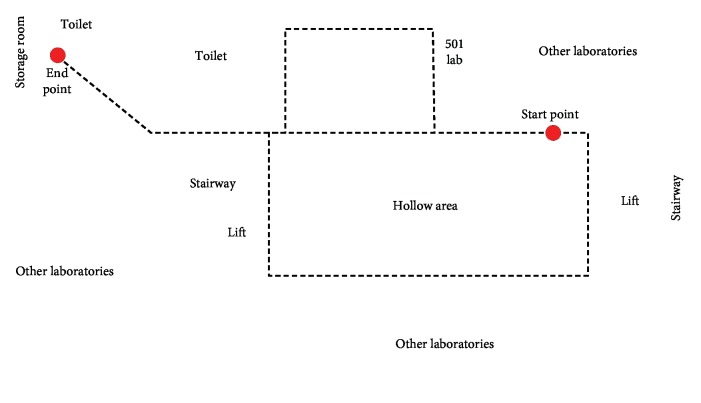
The trajectory of the robot in the indoor environment.

To test the basic performance of the proposed approach, a semantic map building experiment is conducted, where the environment is a comprehensive laboratory building at our university (Figure 7). The main scenes are shown in Figures 6(f)–6(h), including laboratory, toilet, and corridor, which are called as 501 Lab, Toilet, and Corridor, respectively. In this experiment, 1620 images of the three scenes are used to train the ResNet neural network, and the training time used is 738.324(s). During the semantic map building, 2420 frames of the continuous scenes are used and 362 key frames are obtained. The total computation time of the semantic SLAM system in the indoor experiment is 462.472(s).

**Figure 8:**
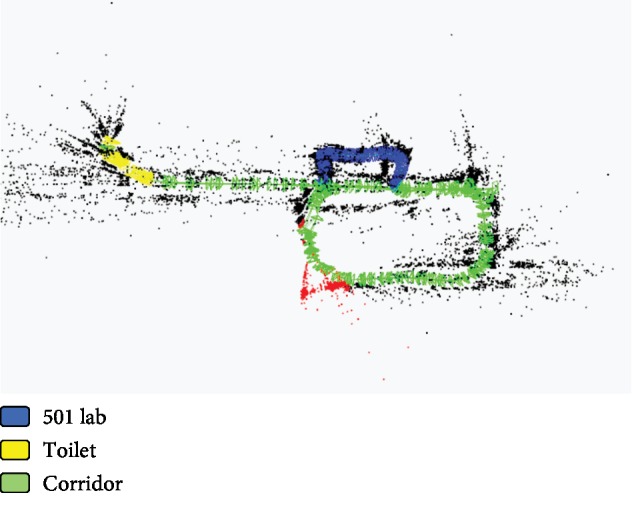
The indoor experimental results of the semantic SLAM task.

The experimental results are shown in Figure 8 and Table 1. In Figure 8, the blue, yellow, and green colors denote the scenes of 501 Lab, Toilet, and Corridor, respectively, which are based on the classification results on the key frames. The red and black points mean the cloud points obtained during the process of the ORB-SLAM, and the red color denotes the cloud points at current observation time.

The results in Table 1 show that the average success rate of scene recognition can reach more than 90% based on the proposed method. In the indoor semantic map building process, the main difficulty is how to deal with the transition of different scenes. The results of the semantic map in Figure 8 show that the proposed method can deal with this problem very well based on the proposed activity value judgment strategy. The results in Figure 8 show that Toilet is misidentified as Corridor at some points. The main reason is that there some similar areas between Toilet and Corridor in the test environment, such as the ground and wall area without other objects. The experimental results prove the method proposed in this paper is effective and feasible for robot semantic map building in complex indoor environments.

### 3.2. The Experiment of Mix Environment

**Figure 9:**
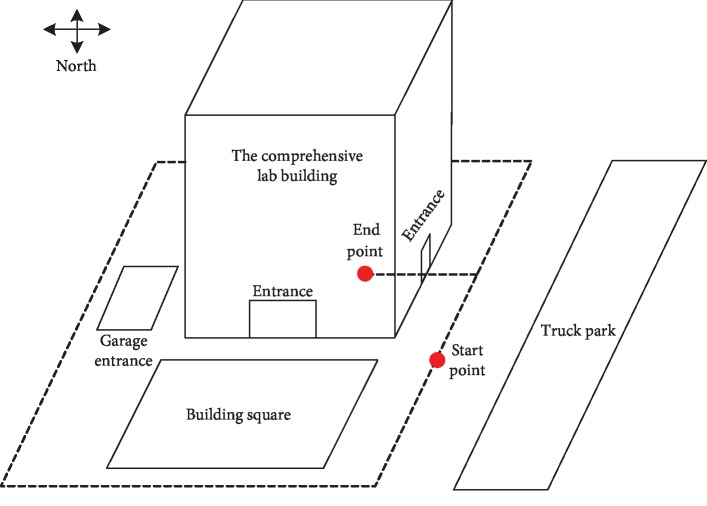
The trajectory of the robot in the mix environment.

**Figure 10:**
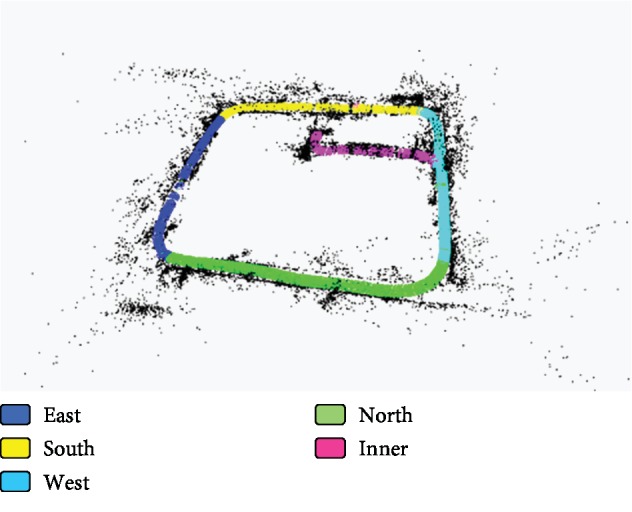
The experimental results of the semantic SLAM task in the mix environment.

To further test the proposed semantic SLAM method, an experiment is conducted, where the environment includes the circle around the comprehensive lab building and the internal part of this building (Figure 9). The main scenes are shown in Figures 6(a)–6(e), including the corridor, carport, residential building, bicycles, and cars. These scenes are abstracted as five semantic labels, namely, east of the lab building (EAST), south of the lab building (SOUTH), west of the lab building (WEST), north of the lab building (NORTH), and the internal passage of the lab (INNER). The experimental results are shown in Figure 10 and Table 2. The five scenes are denoted by different colors. In this experiment, 2700 images are used to train the ResNet and 5570 frames of the continuous scenes are used to build the semantic map. And, the number of the key frames obtained is 906. The training time for the ResNet network and the map building time of the semantic SLAM system are 1205.841(s) and 1050.347(s), respectively.

The results of Table 2 show that the accuracy about WEST and NORTH is less than that of the other categories, and the main reason is that there are not too much prominent features in these two scenes compared with other three scenes, such as parking spaces, bicycle parking areas, and notice bars (Figure 6). However, the accuracy of the two scenes based on the proposed ResNet is high enough for the robot semantic SLAM system. In addition, the results of this experiment show that some recognition rates of the outdoor environment are lower than the indoor environment, the main reason is that there are big turn angles in the robot movement under the outdoor environment. But, the total recognition of the proposed approach in this complex mix environment is high enough, because the continuity determination mechanism of key frame activity value is introduced in the proposed approach. This determination mechanism can recognize the continuous key frame images as the current category before completing the turn, which effectively reduces the error rate of semantic map (Table 2 and Figure 10).

## 4. Discussion

The experiments in Section 3 show that the proposed approach can complete the semantic SLAM task for robot effectively. In this section, the performances of the proposed approach are discussed on the key improvement part of the proposed approach, including image matching algorithm and the monocular visual SLAM algorithm.

**Figure 11:**
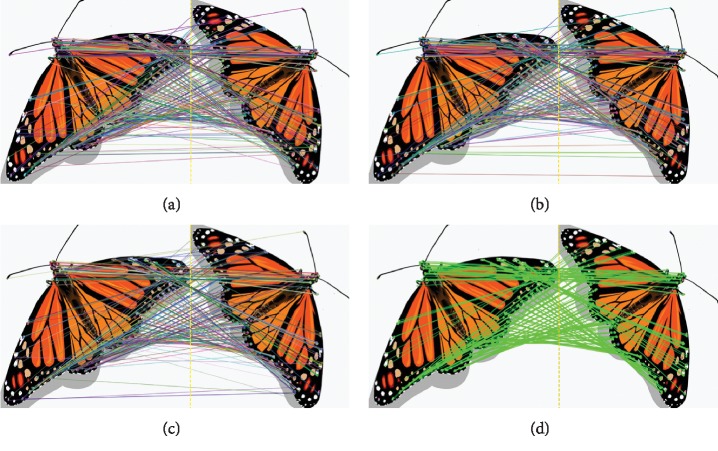
The image matching results of two same images with different view angles based on (a) SIFT, (b) SURF, (c) G-ORB, and (d) and I-ORB.

**Figure 12:**
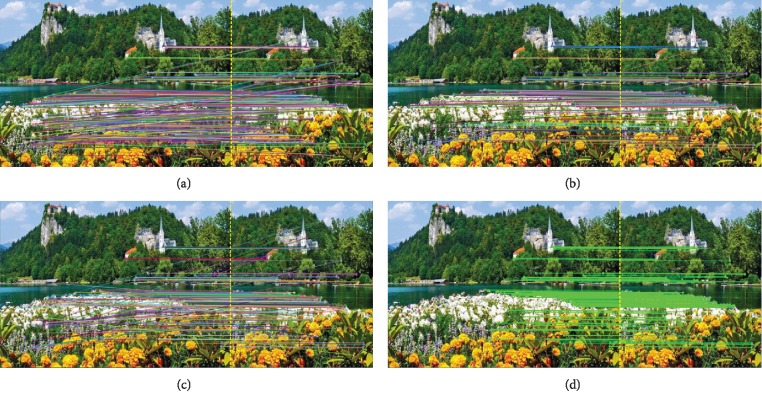
The image matching results between a complete scene image and a part of the same scene image based on (a) SIFT, (b) SURF, (c) G-ORB, and (d) I-ORB.

Firstly, the performance of the improved ORB image matching algorithm (I-ORB) is discussed, by a comparative experiment with the general ORB (G-ORB) algorithm, the general SURF-based algorithm, and the general SIFT-based algorithm. The experiment was carried out on the laptop with the same configuration used in Section 3 and realized by programming on the platform of Microsoft Visual Studio 2012. The results are shown in Figures 11 and 12 and Table 3.  Figure 11 is the image matching experimental results of two same images with different view angles.  Figure 12 is the image matching results between the complete scene image and the partial scene image. Table 3 is the quantitative evaluation of the four algorithms on 10 different image matching experiments. The results show that the general ORB-based method has a good performance on computation speed but has low matching accuracy. The SIFT and SURF algorithms both have good performance on the matching accuracy. And, the proposed I-ORB algorithm has both high matching accuracy and fast computation speed (Table 3), and the main reasons are the improved algorithm of this paper can make sure the number of feature points and effectively eliminate the false matching feature points at the same time.

**Figure 13:**
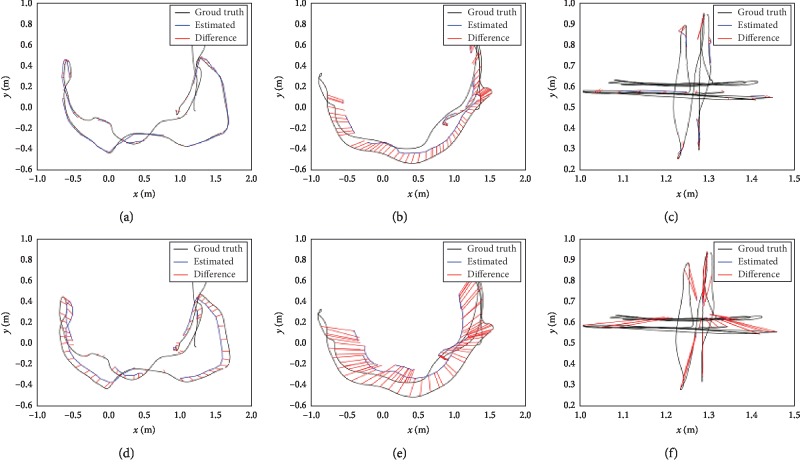
The SLAM experimental results on (a) fre1_desk1 based on I-ORB, (b) fre1_desk2 based on I-ORB, (c) fre1_xyz based on I-ORB, (d) fre1_desk1 based on G-ORB, (e) fre1_desk2 based on G-ORB, and (f) fre1_xyz based on G-ORB.

Another important part of the proposed approach is the monocular visual SLAM algorithm, which is the basis of the semantic SLAM algorithm. To discuss the performance of the improved ORB-based visual SLAM algorithm (I-ORB), a comparative experiment with the general ORB-based visual SLAM algorithm (G-ORB) is conducted. And, the widely used data set TUM is selected to test the performance of the two algorithms [38, 39]. Three subsets of TUM (fre1_desk1, fre1_desk2, and fre1_xyz) are selected to conduct five experiments on the two algorithms, respectively. The trajectory lengths of fre1_desk1, fre1_desk2, and fre1_xyz are 9.62 m, 10.16 m, and 7.11 m, respectively. All the frame rates of the three subsets are 30 fps. Root-mean-square error (RMSE) is used to evaluate the two SLAM methods:

(9) $$\begin{array}{c} \text{RMSE}=\sqrt{\frac{1}{z}\overset{z}{\underset{i=1}{\sum }}{\left(X_{i}-X_{i}^{\prime }\right)}^{2}}, \end{array}$$

where *X*_*i*_ is the coordinate estimation value of robot at the time *i*, *X*_*i*_′ is the real position coordinate of robot at the time *i*, and *z* is the number of the total time in the experiment. The experimental results are shown in Figure 13 and Table 4.

Table 4 shows that the proposed approach can obtain map cloud points and key frame images in the three data sets more efficiently than the general ORB-based method under the same experimental conditions. The results of the fre1_desk1 show that the experimental track of the proposed algorithm in this paper basically coincides with the real track, while the error of the traditional algorithm is relatively obvious (Figures 13(a) and 13(d)). In the experiment of fre1_desk2, there are fast translation motion, rapid rotation motion of the camera, and not obvious features of scenes in the rotation process. The results of this data set show that the error increased based on both the two algorithms. However, the algorithm presented in this paper shows better stability when facing the camera's fast translation and rotation motion and obtains more key frames than the traditional algorithm. And, the track error of the proposed algorithm is relatively small (Figures 13(b) and 13(e)). The results of fre1_xyz that is characterized by a small range of movement and relatively slow speed, almost no rotation movement, and also it shows that the proposed approach is more efficient than the general ORB-based SLAM algorithm (Figures 13(c) and 13(f)).

## 5. Conclusion

In this paper, an improved semantic SLAM algorithm for monocular robot has been presented. In the proposed approach, an improved method of image feature points matching is presented firstly. Then, an improved robot visual SLAM system is proposed, to deal with the problem of insufficient capacity, low computation, and positioning accuracy in the traditional visual SLAM method. Finally, an improved semantic map building method is proposed by combining the semantic labels obtained by ResNet residual network with the visual SLAM method. Experiments are carried out in real scenes, and the experimental results prove that the algorithm in this paper is effective for robot semantic SLAM task. In addition, some comparisons are made on the performances of the proposed approach with the general methods, and the results prove that the proposed approach has better implementation effects than traditional algorithms in image matching and visual SLAM tasks. In future work, some large scale and more complex semantic SLAM tasks will be further studied.

## Figures and Tables

**Table 1:** The experimental results of semantic recognition in the indoor environment.

Semantic labels	Total number of scenes	False identification of scenes	Recognition accuracy (%)
501 lab	65	3	95.38
Corridor	200	15	92.50
Toilet	35	5	85.71
Average value	100	7.67	92.33

**Table 2:** The experimental results of semantic recognition in the mix environment.

Semantic labels	Total number of scenes	False identification of scenes	Recognition accuracy (%)
West	80	10	87.50
South	80	6	92.50
East	80	5	93.50
North	80	8	90.00
Inner	80	0	100.00
Average value	80	5.8	92.70

**Table 3:** The valuation of the four methods for image matching experiments.

Algorithms	Matching accuracy (%)	Computation time (s)
SIFT	85.5	5.14
SURF	90.5	0.96
G-ORB	56.6	0.42
I-ORB	99.8	0.68

**Table 4:** The valuation of the two methods for SLAM tasks on various data sets.

Date set	Number of key frames		Number of map points		RMSE	
	G-ORB	I-ORB	G-ORB	I-ORB	G-ORB	I-ORB
fre1_desk1	68	79	3157	4129	0.075	0.019
fre1_desk2	121	138	4236	5714	0.303	0.140
fre1_xyz	31	33	1714	1827	0.168	0.032

## Data Availability

The data supporting this study are openly available from the website at https://vision.in.tum.de/data/datasets/rgbd-dataset/download.
